# Circumscription and Phylogenetic Position of Two Propagulose Species of *Syntrichia* (Pottiaceae, Bryophyta) Reveals Minor Realignments within the Tribe Syntricheae

**DOI:** 10.3390/plants11050626

**Published:** 2022-02-25

**Authors:** M. Teresa Gallego, María J. Cano, Juan A. Jiménez, Juan Guerra

**Affiliations:** Departamento de Biología Vegetal (Botánica), Facultad de Biología, Universidad de Murcia, Campus de Espinardo, 30100 Murcia, Spain; mcano@um.es (M.J.C.); jajimene@um.es (J.A.J.); jguerra@um.es (J.G.)

**Keywords:** ITS, Pottiaceae, *Sagenotortula*, *Streptopogon*, *Syntrichia*, *S. amphidiacea*, *S. gemmascens*, taxonomy, *trn*G, *trn*L-F

## Abstract

In the course of a worldwide revision of the genus *Syntrichia*, we identified problems in the circumscription of some species of the genus as well as among some allied genera grouped in the tribe Syntricheae. This is the case for the two propagulose *Syntrichia amphidiacea* and *S. gemmascens*, closely related to *Streptopogon.* We analyzed phylogenetic relationships between these species, based on nuclear (ITS) and two plastid (*trn*L-F and *trn*G) markers and morphological features. Species delimitation using molecular data was consistent with our preliminary morphological inference. Phylogenetic analyses were performed using maximum likelihood and Bayesian inference methods. Our results placed *Syntrichia amphidiacea* in the *Streptopogon* clade. *Syntrichia gemmascens* is also included in *Streptopogon* in spite of the discrepancy of the ITS and plastid relationships, which could be evidence of an exchange of genetic material between species in various lineages in the Pottioideae. *Streptopogon* is maintained as a separate genus on the basis of morphology characters, and we consider the differentiation of laminal papillae and the presence of a stem central strand as new characters in the genus. We accept *Sagenotortula* as distinct genus sister to *Syntrichia*. We consider the lack of costal dorsal epidermis and the differentiation of a crescent-shaped costal dorsal stereid band as distinctive generic characters in *Syntrichia.* Additionally, we include *Syntrichia percarnosa* as a new synonym for *S. breviseta*. Three names are lectotypified.

## 1. Introduction

The Pottiaceae, characteristic of harsh habitats [1], are one of the most complex and diverse family of mosses and are widely distributed around the world [2,3,4,5]. The taxonomy of Pottiaceae has been complicated and its generic circumscriptions are the subject of ongoing debate [6]. The genus *Syntrichia* Brid. is one of the most diverse genera within the family, with about 90 species currently known with a focus of diversification in South America [7,8]. Zander [3,9] and Ochyra [10] established its morphological characterization and distinguished it from *Tortula* Hedw., focusing the weight of its generic differentiation on the anatomy of the costa. A few years later, Spagnuolo et al. [11] supported this segregation with molecular data. At the molecular level, only a few phylogenetic studies on *Syntrichia* have been conducted to date, all with a limited number of species [12,13,14,15].

**Figure 1:**
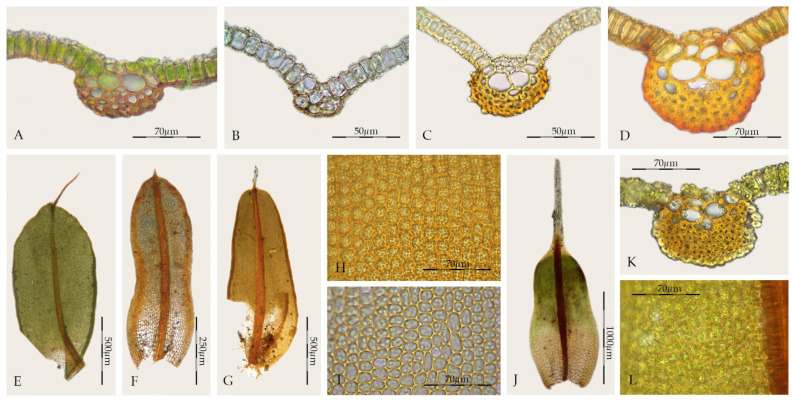
Morphological characters in *Syntrichia* and *Willia*: (**A**) *Syntrichia* *percarnosa*, cross-section of the costa at the middle of the leaf (MUB 40305); (**B**) *S. norvegica*, cross-section of the costa in the upper third of the leaf (MUB 8345); (**C**) *S. ruralis*, cross-section of the costa at the middle of the leaf (MUB 28269); (**D**) *S. serripungens*, cross-section of the costa at the middle of the leaf (MUB 40474); (**E**) *S. norvegica*, stem leaf (MUB 8345); (**F**) *S. costesii*, stem leaf (MUB 38288); (**G**) *S. ruralis*, stem leaf (MUB 28269); (**H**) *S. fragilis*, middle laminal cells (MUB 23105); (**I**) *S. amphidiacea*, middle laminal cells (MUB 56123); (**J**) *Willia austroleucophaea*, stem leaf (MUB 60670); (**K**) *W. brachychaete*, cross-section of the costa at the middle of the leaf (MUB 17946); (**L**) *W. brachychaete*, middle laminal cells (MUB 17946). Photos: by M. Teresa Gallego.

*Syntrichia* is a heterogeneous genus characterized by the costa having a dorsal stereid band that is usually crescent-shaped in cross-section and lacking a dorsal epidermis (Figure 1A–D), laminal cells red with KOH, basal cells differentiated and forming a hyaline area on each side of the costa, exserted sporophytes, perichaetial leaves usually undifferentiated, peristome differentiated, and the calyptra cucullate and smooth. Other characters, leaf shape, marginal curvature, differentiation of a border (Figure 1E–G), marginal teeth near the leaf apex, laminal stratification, hair-point, size and papillosity of dorsal costal cells, and the differentiation of a sclerodermis, hyalodermis, and stem central strand, are extremely variable characters in the genus. Leaves with strongly papillose laminal cells also characterize *Syntrichia*, as well as the high variability of the number of papillae per cell, as well as the shape or the arrangement of the papillae (Figure 1H,I). The delimitation of the paracostal basal cells from those of the rest of the lamina is distinctive in *Syntrichia* in shape (two inverted “U” or like a horns) and length, although some species within the genus have a poorly differentiated hyaline basal area of the leaf.

Zander [3], in the most comprehensive monograph of Pottiaceae genera, proposed the currently used circumscription for *Syntrichia*, which he updated a few years later [1]. Species circumscriptions have also undergone changes since the second half of the 20th century [3,7,14,16,17,18,19,20,21,22]. Recently, Brinda et al. [23] have proposed an infrageneric classification for *Syntrichia* on the basis of an unpublished molecular investigation for establishing the new names to be used in forthcoming publications. These authors propose a broad circumscription of the genus including most of the closely related genera, considering nine sections: the type section of *Syntrichia*; four sections to include *Calyptopogon* (Mitt.) Broth., *Sagenotortula* R.H. Zander, *Willia* Müll. Hal., and *Streptopogon* Wilson ex Mitt., which includes *Syntrichia* sect. *Collotortula* R.H. Zander [3], respectively; two new sections for species with lanceolate and denticulate margins, sheathing bases and without elongated hair-points (sect. *Magnisyntrichia* Brinda, Jáuregui-Lazo & Mishler and sect. *Eosyntrichia* Brinda, Jáuregui-Lazo & Mishler); the sect. *Aesiotortula* R.H. Zander which has been retained but with a different circumscription from that designated by Zander [3], and the sect. *Vallidens* (Müll. Hal.) Brinda, Jáuregui-Lazo & Mishler for the small species with plane leaf margins, a short proportion of differentiated basal cells in relation to leaf length, and occasionally modified, caducous leaf apices. This classification represents a considerable realignment within the potentially monophyletic genus. This situation will most likely be proposed in the subsequent work mentioned by Brinda et al. [23]. With all this research, the taxonomy of *Syntrichia* and its allied genera becomes tremendously exciting, encouraging new collections and studies in Pottioideae. According to Cano et al. [6], most genera included in the Pottiaceae have not been the subject of extensive study using molecular data, and part of the studied genera have been resolved as paraphyletic or polyphyletic molecular entities. This situation can be assumed to be advantageous, as molecular paraphyly provides important information about evolutionary processes and, therefore, should not be suppressed by phylogenetic practices to preserve strict monophyly [24].

Molecular techniques have complemented morphological observations traditionally used in taxonomy. The result is undoubtedly a more efficient and more resolute integrative taxonomy, which in turn is compiled in valuable databases. On the other hand, botanical field work provides an important part of the observations necessary for a better understanding of taxa, and provides the main source of information for the advancement of systematics and knowledge of biodiversity.

During many years working on the worldwide revision of the genus *Syntrichia* and after studying thousands of specimens, we have been able to verify the richness and variability of the phenotypic features that traditionally differentiate the species of this genus, as well as the difficulty in establishing taxonomically valuable characters. Through morphological observation and the study of the habitat and distribution of the South American Pottiaceae, carried out by the research group [25], we have been able to identify problems in the circumscription of some species of *Syntrichia*, as well as among the related genera grouped in the tribe Syntricheae sensu Zander [1]. It has been the case for the widespread *Syntrichia amphidiacea* (Müll. Hal.) R.H. Zander and the Chinese *S. gemmascens* (P.C. Chen) R.H. Zander, two closely related taxa to *Streptopogon* which share many morphological, anatomical, and ecological characters. Both species have been recently included in *Syntrichia* sect. *Streptopogon* (Wilson ex Mitt.) Brinda, Jáuregui-Lazo & Mishler by Brinda et al. [23].

**Figure 2:**
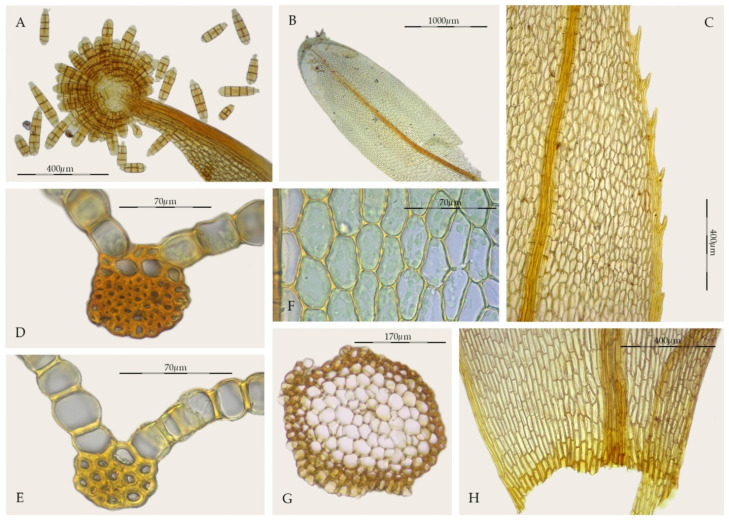
Morphological characters in *Streptopogon*: (**A**) *Streptopogon calymperes* propagulose leaf apex (MUB 43082); (**B**) *S. cavifolius*, stem leaf (NY 00598442); (**C**) *S. calymperes*, detail of the toothed leaf margin in the upper third (MUB 32954); (**D**) *S. calymperes*, cross-section of the costa at the middle of the leaf (MUB 43082); (**E**) *S. calymperes*, cross-section of the costa at the middle of the leaf (MUB 32954); (**F**) *S. calymperes*, middle laminal cells (MUB 43082); (**G**) *S. calymperes*, cross-section of the stem (MUB 32954); (**H**) *S. calymperes*, leaf base (MUB 32954). Photos: by M. Teresa Gallego.

**Figure 3:**
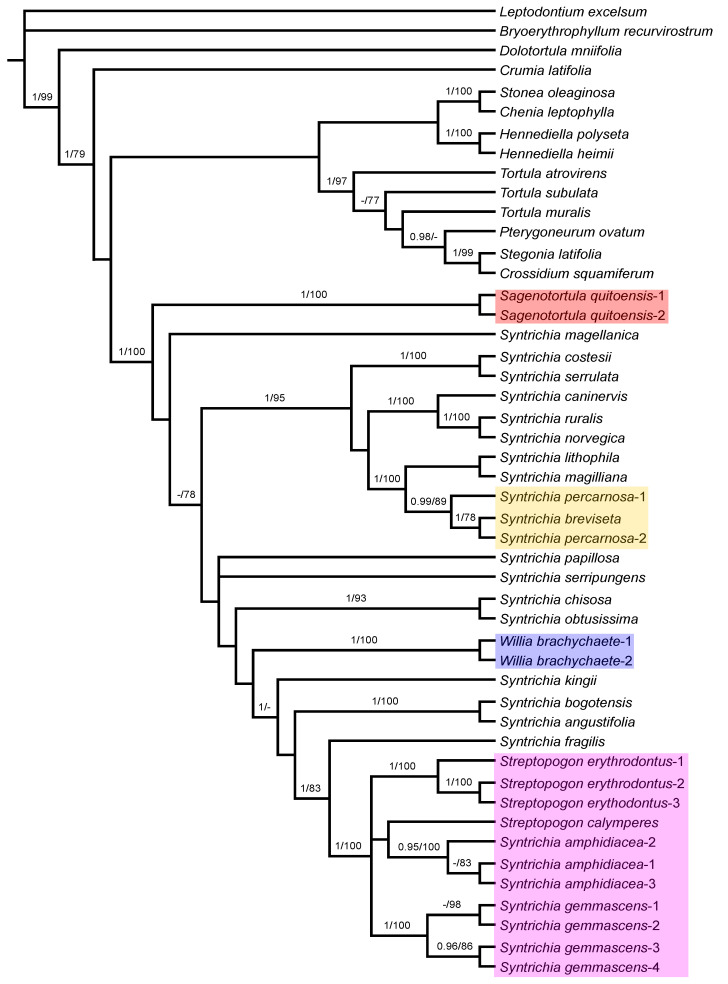
Majority-rule consensus tree of the Bayesian inference analysis inferred from ITS sequences. Bayesian posterior probabilities (PP), followed by maximum likelihood bootstrap values (BS) are shown above the branches. Support values of BS < 70 and PP < 0.95 are not shown. The highlighted clades include species considered in *Sagenotortula*, *Streptopogon*, *Syntrichia*, or *Willia* in this study and are referred to in the text.

**Figure 4:**
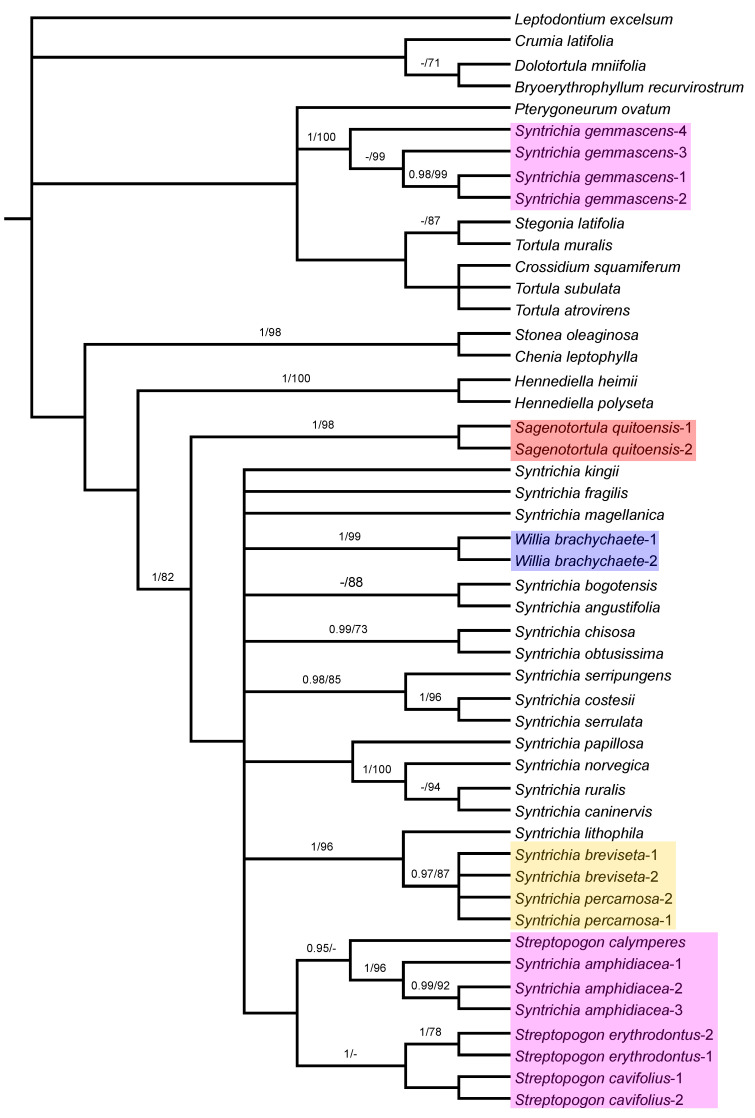
Majority-rule consensus tree of the Bayesian inference analysis inferred from combined plastid (*trn*G and *trn*L-F) sequences. Bayesian posterior probabilities (PP), followed by maximum likelihood bootstrap values (BS) are shown above the branches. Support values of BS < 70 and PP < 0.95 are not shown. The highlighted clades include species considered in *Sagenotortula*, *Streptopogon*, *Syntrichia*, or *Willia* in this study and are referred to in the text.

**Figure 5:**
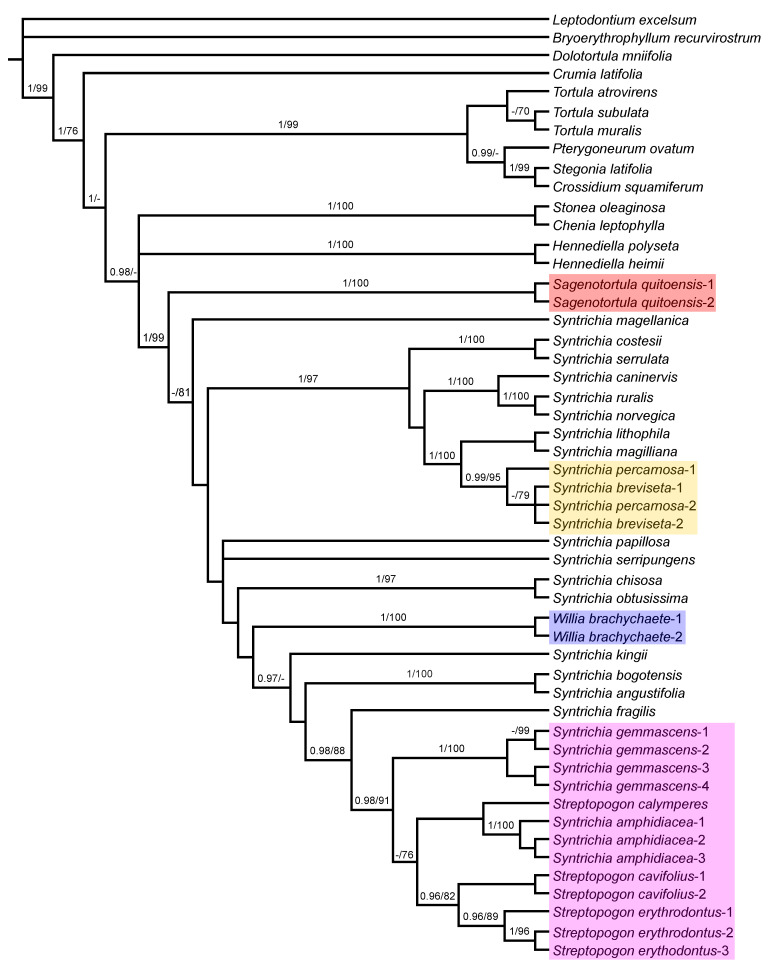
Majority-rule consensus tree of the Bayesian inference analysis inferred from combined plastid (*trn*G and *trn*L-F) and ITS sequences. Bayesian posterior probabilities (PP), followed by maximum likelihood bootstrap values (BS) are shown above the branches. Support values of BS < 70 and PP < 0.95 are not shown. The highlighted clades include species considered in *Sagenotortula*, *Streptopogon*, *Syntrichia*, or *Willia* in this study and are referred to in the text.

**Figure 6:**
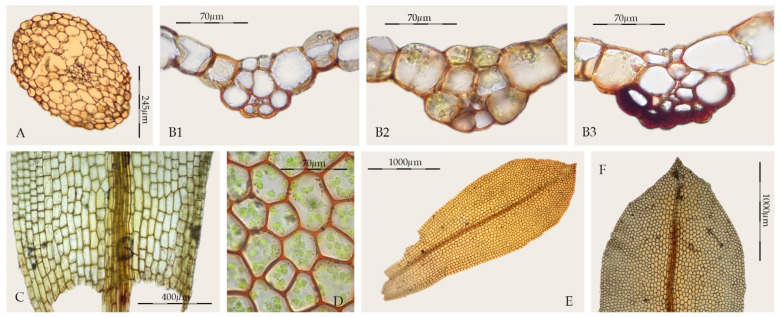
*Sagenotortula quitoensis* (MUB 23065): (**A**) Cross-section of the stem; (**B**) Cross-section of the costa, 1: at the upper third of the leaf, 2: at the middle of the leaf, 3: at the lower third of the leaf; (**C**) Base of the leaf; (**D**) Middle laminal cells; (**E**) Stem leaf; (**F**) Leaf apex. Photos: by M. Teresa Gallego.

*Streptopogon* is a small genus of Pottiaceae with a Neotropical to Paleotropical distribution and seven species are currently recognized [26,27]. *Streptopogon calymperes* Müll. Hal. is the type of the genus. According to Casado [26], the generic characters that unify *Streptopogon* include: (1) scabrous and mitrate calyptrae, (2) short and twisted setae, (3) exserted or emergent, wide, and cylindrical capsules, (4) absence of a stem central strand (Figure 2G), (5) a strong costa with a single stereid band (Figure 2D,E), and (6) smooth leaf cells (Figure 2F). Other distinguishing characteristics of the genus according to Zander [3] are: (1) laminal KOH reaction usually red, (2) basal cells little differentiated from the upper cells (Figure 2H), (3) the presence of clavate propagula on leaves or costae (Figure 2A,B), (4) leaves entire to denticulate or serrate in the upper third (Figure 2C), (5) lack of hydroids in the costa, (6) transverse section of the costa with the dorsal stereid band round to semicircular, (7) costal dorsal epidermis present or occasionally absent (Figure 2D), and (8) perichaetial leaves not or little sheathing. Salmon [28], in his excellent monograph on *Streptopogon* emphasized its similarity to *Syntrichia*, but highlighted its generic differentiation on the basis of the smooth laminal cells and mitrate and scabrous calyptrae, although pointing out that some species of *Streptopogon* have glabrous calyptra. The placement of this genus in the family Pottiaceae is mainly supported by the haplolepidous, filamentous, and twisted peristome [3,26,28], resembling those of *Syntrichia*.

With the intention of circumscribing the two propagulose species, *Syntrichia amphidiacea* and *S. gemmascens*, that are closely related to *Streptopogon*, we here present a phylogenetic study of *Syntrichia* and its allied genera (*Sagenotortula*, *Streptopogon*, and *Willia*) on the basis of nuclear (ITS) and two plastid (*trn*L-F and *trn*G) markers, and a morphological study to (1) investigate if the molecular result agrees with prior morphological studies, (2) investigate the phylogenetic relationship of *Syntrichia* and *Streptopogon* within Pottioideae, and (3) determine their phenotypic differentiation, updating morphological distinctive characters at the generic level, and providing the morphological basis for differentiating taxa.

## 2. Materials and Methods

### 2.1. Morphological Study

For morphological delimitation, taxonomic conclusions and characterization as part of the work to develop a worldwide revision of *Syntrichia*, we studied specimens from these herbaria: B, BA, BM, BOLUS, BR, CANM, CAS, COLO, CU, DUKE, E, EGR, F, FH, FI, FLAS, FT, GB, H, JE, L, LIL, LPB, M, MA, MEXU, MICH, MO, MUB, NMW, NY, O, PC, PRE, S, SGO, SP, U, UPS, US, W, and Z, as well as material collected in the field by the authors [25]. Additionally, most of the type material of the taxa currently attributed to *Syntrichia* from throughout the world was studied. The morphological study of the allied genera has been complemented by the examination of most of their original material. We used the conventional anatomical and morphological methods applied for the Pottiaceae [3]. Microscopic examinations and measurements were taken with an Olympus-BH2 light microscope, while microphotographs were obtained with a Jenoptik ProgRes C7 camera mounted on this microscope.

### 2.2. Molecular Taxon Sampling

To understand the position of *S. amphidiacea* and *S. gemmascens* in relation to other members of the genus, we included 1–4 specimens of: *Syntrichia amphidiacea* (3), *S. angustifolia* (Herzog) M.J. Cano (1), *S. bogotensis* (Hampe) Mitt. ex R.H. Zander (1), *S. breviseta* (Mont.) M.J. Cano & M.T. Gallego (2), *S. caninervis* Mitt. (1), *S. chisosa* (Magill., Delgad. & L.R. Stark) R.H. Zander (1), *S. costesii* (Thér.) R.H. Zander (1), *S. fragilis* (Taylor) Ochyra (1), *S. gemmascens* (4), *S. kingii* (H. Rob.) M.T. Gallego & M.J. Cano (1), *S. lithophila* (Dusén) Ochyra & R.H. Zander (1), *S. magellanica* (Mont.) R.H. Zander (1), *S. magilliana* L.E. Anderson (1), *S. norvegica* F. Weber (1), *S. obtusissima* (Müll. Hal.) R.H. Zander (1), *S. papillosa* (Wilson ex Spruce) Spruce (1), *S. percarnosa* (Müll. Hal.) R.H. Zander (2), *S. ruralis* (Hedw.) F. Weber & D. Mohr (1), *S. serripungens* (Lorentz & Müll. Hal.) R.H. Zander (1) and *S. serrulata* Warnst. (1). For the outgroup, we included eight members of the Syntricheae to cover the variation of the tribe (1–3 specimens per species): *Chenia leptophylla* (Müll. Hal.) R.H. Zander (1), *Crumia latifolia* (Kindb.) V.D. Schofield (1), *Dolotortula mniifolia* (Sull.) R.H. Zander (1), *Hennediella heimii* (Hedw.) R.H. Zander (1), *H. polyseta* (Müll. Hal.) R.H. Zander (1), *Sagenotortula quitoensis* (Taylor) R.H. Zander (2), *Stonea oleaginosa* (I.G. Stone) R.H. Zander (1), *Streptopogon calymperes* (1), *S. cavifolius* Mitt. (2), *S. erythrodontus* (Taylor) Wilson ex Mitt. (3) and *Willia brachychaete* (Dusén) R.H. Zander (2); also, we selected some more distantly related Pottiaceae members according to Cano et al. [6] and Jiménez et al. [29], including four representative Pottieae sensu Zander [1]: *Bryoerythrophyllum recurvirostrum* (Hedw.) P.C. Chen (1), *Crossidium squamiferum* (Viv.) Jur. (1), *Pterygoneurum ovatum* (Hedw.) Dixon (1), *Stegonia latifolia* (Schwägr.) Venturi ex Broth. (1), *Tortula atrovirens* (Sm.) Lindb. (1), *T. muralis* Hedw. (1) and *T. subulata* Hedw. (1). *Leptodontium excelsum* (Sull.) E. Britton was used to root the phylogeny to represent the sister lineage as well.

All sequences were newly generated for these analyses, except for 58 sequences that were downloaded from GenBank and published previously by us. Moreover, DNA of 7 specimens already sequenced for plastid loci in Gallego et al. [14] and submitted to GenBank were amplified for the nuclear loci. Specimens are provided in Appendix A, including information on locality, herbarium references, and GenBank accession numbers.

### 2.3. DNA Extraction, Amplification, and Sequencing

Total genomic DNA from the distal portion of a few dried gametophores per specimen was extracted using the CTAB protocol [30] or the protocol for extraction by Suzuki et al. [31] and stored at −20 °C until the polymerase chain reaction (PCR) was carried out. We selected three loci: two from the chloroplast genome, the *trn*GUCC G2 intron (*trn*G), and the *trn*LUAA exon *trn*FGAA region (*trn*L-F), as well as the nuclear internal transcribed spacers 1 and 2 (ITS1-5.8SITS2). The ITS1 and ITS2 were either amplified and sequenced separately or in a single amplification. These loci have been shown to be useful for phylogenetic reconstruction in the Pottiaceae [6,14,29,32]. The primer pairs used for each locus were *trn*G-F/*trn*G-R [33], *trn*C/*trn*F [34], ITS5-bryo/ITS4-bryo [35], ITS1-F/ITS1-R [36], and seqITS2 [37].

Amplification reactions were performed using an Eppendorf Mastercycler in a 25 μL volume containing 1 μL Taq DNA Polymerase (1 U/μL; Biotools, Madrid, Spain), 2.5 μL of Mg^2+^ buffer provided by the manufacturer, 2 μL of 2.5 mM dNTP mix, 1.5 μL of each primer (10 μM), and 1 μL of the DNA extract. Thermocycling conditions for the *trn*G and *trn*L-F were: 94 °C for 5 min linked to 34 cycles at 94 °C for 30 s, 52 °C for 30 s, and 72 °C for 1 min with a final extension of 72 °C for 7 min. The amplification cycle for nrITS was: 94 °C for 4 min, followed by 40 cycles at 94 °C for 1 min, 52 °C for 30 s, and 72 °C for 1 min, and a final extension step at 72 °C for 10 min. Finally, 2 μL of the amplification products were visualized on 1.5% agarose gel and successful amplifications were purified using the GenElute PCR Clean-Up kit (Sigma-Aldrich, St. Louis, MO, USA), and sequenced at Macrogen Spain (Madrid, Spain). Nucleotide sequence contigs were edited and assembled for each DNA region in Geneious 9.1.8 [38]. Consensus sequences were aligned using default parameters of MUSCLE [39] implemented in Geneious with minor manual adjustments in a few sections. Regions of partially incomplete data in the beginning and end of the sequences were identified and were excluded from subsequent analyses. Indels were coded using SeqState v.1.4.1 [40] using the simple indel coding model as suggested by Simmons and Ochoterena [41]. We present the analyses with the indels included since these provided additional phylogenetic evidence. Each gene partition was tested for the best-fit substitution model using jModelTest v.2.1.6 [42] under the Akaike information criterion (AIC) and Bayesian information criterion (BIC). The selected model was TrN+I+G [43] for *trn*G, *trn*L-F, and nrITS.

### 2.4. Phylogenetic Analysis Sequencing

Phylogenetic relationships were analyzed using both maximum likelihood (ML) and Bayesian inference (BI). Analyses were performed separately on each data set and the chloroplast data were combined afterward. To check for incongruence among the plastid and nrITS datasets, phylogenetic reconstructions under ML and BI were visually compared. The node bootstrap support of ≥70 in the ML analysis and posterior probability ≥0.95 were chosen as values for supported incongruence.

Maximum likelihood analyses were performed using RAxML [44] through the graphical font-end raxmlGUI v.2.0 [45]. A rapid bootstrap option with 1000 replicates and search for the best-scoring ML tree were conducted under the GTRCAT model for all concatenated and individual gene data sets. Nodes with bootstrap (BS) values equal to or above 70% were treated as well supported.

Bayesian inference analyses (BI) were performed using MrBayes v.3.2.6 [46] on the CIPRES Gateway v.3.3 [47], running a partitioned analysis and specifying a substitution model for each block. The data were analyzed using Markov chain Monte Carlo (MCMC), running two parallel analyses with four chains each for 5 million generations, sampling trees and parameters every 1000 generations. Twenty-five percent of the tree was discarded as burn-in. The resulting trees for both ML and BI analyses were visualized and partially edited in FigTree v.1.4.4 [48]. Posterior probability (PP) of 0.95–1.00 were considered to be strong support.

## 3. Results

### 3.1. Phylogenetic Analyses

We generated 82 new sequences. We obtained sequences for the three loci for all specimens with the exception of ITS1 and ITS2 spacers for *Syntrichia breviseta*-2 and *Streptopogon cavifolius*-1 and 2, ITS1 spacer for *Syntrichia gemmascens*-4, ITS2 spacer for *Streptopogon calymperes* and *S. erythrodontus*-3, *trn*G spacer for *Pterygoneurum ovatum*, *Sagenotortula quitoensis*-1, *Stegonia latifolia*, *Streptopogon cavifolius*-2, *S. erythrodontus*-3, *Syntrichia lithophila* and *S. magellanica*, *trn*L-F spacer for *Streptopogon cavifolius*-1 and *S. erythrodontus*-3, and finally, *trn*G and *trn*L-F spacers for *Syntrichia magilliana*. For the final analyses, we created two datasets: the chloroplast and nrITS databases. Summary characteristics of each dataset are presented in Table 1. As expected, the chloroplast loci contributed less phylogenetic information than ITS. The ML and BI analyses of each individual marker had nearly identical topologies. Therefore, only the Bayesian inference topologies are shown here (Figure 3, Figure 4 and Figure 5), with bootstrap support (BS) as well as posterior probabilities (PP) values added where applicable. The topology of the phylogenetic trees of the ITS and the chloroplast sequences were practically identical for the two options of indels, although with some higher support values of main nodes with indels included, thus, they were included as informative in the analyses. The resulting trees based on combined plastids and nuclear sequences revealed the position of *Syntrichia gemmascens* as the unique conflict between partially well-supported structures (Figure 3 and Figure 4). As there were no major differences in the topologies of all obtained trees, with the exception of a single case of incongruence indicated above, the plastid and nuclear data were concatenated. The relationships between the species currently considered in the tribe Syntricheae according to Zander [1] are not resolved. Nuclear analyses support *Syntrichia* as monophyletic only with *Sagenotortula*, *Willia*, and *Streptopogon* included. Plastid analyses only support the monophyly of *Syntrichia* if *S. gemmascens* is excluded from the genus. Finally, plastid and nuclear combined analysis support a similar result than the ITS-derived tree.

### 3.2. Analysis of ITS Sequences

The phylogenetic tree based on ITS sequences (Figure 3) shows a polytomy with *Bryoerythrophyllum recurvirostrum* and a clade with the remaining accessions currently considered as Pottioideae (PP = 1; BS = 99). At the next level, relationships are poorly resolved and only a few lineages are strongly supported in the Pottioideae: species of *Hennediella* (PP = 1; BS = 100) in an unresolved clade together with other allied Syntricheae genera (*Chenia* and *Stonea*); the Pottieae clade composed by *Tortula atrovirens* sister to other *Tortula* species and the related genera including *Pterygoneurum*, *Stegonia*, and *Crossidium* (PP = 1; BS = 97); *Syntrichia*, *Sagenotortula*, *Willia*, and *Streptopogon* are included in a strongly supported clade (PP = 1; BS = 100). *Streptopogon* clade is strongly supported with *Syntrichia amphidiacea* and *S. gemmascens* included (PP = 1; BS = 100). In both cases, relationships between *Syntrichia* species are poorly resolved and only the next lineages are strongly supported: the clade (PP = 1; BS = 95) that includes the strongly grouped *Syntrichia costesii* and *S. serrulata* (PP = 1; BS = 100) as sister of an unsupported clade including two subclades: the *S. ruralis* group with *Syntrichia caninervis*, *S. ruralis*, and *S. norvegica* (PP = 1; BS = 100), and the strongly supported clade (PP = 1; BS = 100) that accommodates *S. lithophila* and *S. magilliana* as sister to the clade with two specimens of *S. percarnosa* and one sample of *S. breviseta* (PP = 0.99; BS = 89). *Syntrichia chisosa* and *S. obtusissima* are strongly grouped (PP = 1; BS = 93), like *S. bogotensis* and *S. angustifolia* (PP = 1; BS = 100) and two samples of *Willia brachychaete* (PP = 1; BS = 100). The analysis indicates maximum support (PP = 1; BS = 100) for a monophyletic group composed of specimens strongly grouped of *Syntrichia gemmascens* (PP = 1; BS = 100), *S. amphidiacea* (PP = 0.95; BS = 100) as sister to *Streptopogon calymperes* but without support, and *Streptopogon erythrodontus* (PP = 1; BS = 100), with *S. fragilis* as sister (PP = 1; BS = 83).

### 3.3. Analysis of trnG–trnL-F Sequences

The phylogenetic tree derived from combined plastid sequences (Figure 4) shows a polytomy comprised of an unsupported clade with *Crumia latifolia* and a subclade of *Dolotortula mniifolia* and *Bryoerythrophyllum recurvirostrum*, and another clade with the remaining accessions of Pottieae with the strongly supported *Syntrichia gemmascens* nested (PP = 1; BS = 100), but all these relationships are not supported. At the next level, the relationships between the remaining Syntricheae genera (*Chenia*, *Hennediella*, *Stonea*, *Sagenotortula*, *Streptopogon*, *Syntrichia*, and *Willia*) are unresolved. *Syntrichia* is monophyletic with good support (PP = 1; BS = 82), only if *S. gemmascens* is excluded from the genus and the three allied genera (*Sagenotortula*, *Streptopogon*, and *Willia*) are included. *Streptopogon* is monophyletic only with *S. amphidiacea* included, although in an unsupported position.

Within the unresolved clade where the bulk of *Syntrichia* species appear, the relationships between them are generally poorly resolved, and only a few lineages supported: a clade with *S. chisosa* and *S. obtusissima* (PP = 0.99; BS = 73), another clade with *S. serripungens* as sister of *Syntrichia costesii* and *S. serrulata* (PP = 0.98; BS = 85), the *S. ruralis* group with *Syntrichia caninervis*, *S. ruralis*, and *S. norvegica* (PP = 1; BS = 100) with *S. papillosa* as sister in an unsupported clade, and the strongly supported (PP = 1; BS = 96) clade that accommodates *S. lithophila* as sister to the clade of *S. percarnosa* and *S. breviseta*, including *S. percarnosa* and *S. breviseta*. The analysis of plastid sequences indicates an unsupported relationship for the clade composed of three specimens of *Syntrichia amphidiacea* plus *Streptopogon calymperes*, *S. cavifolius*, and *S. erythrodontus*. Only the subclade with all samples of *S. amphidiacea* (PP = 1; BS = 96), sister to *Streptopogon calymperes* (PP = 0.95), and the subclade with the remaining species of *Streptopogon* (PP = 1) are supported.

### 3.4. Analysis of Combined Plastid and ITS Sequences

The overall topology of the combined phylogram (Figure 5) is similar to that of the ITS-derived tree. The strongly supported clade with the accessions currently considered as Pottieae (*Crossidium*, *Pterygoneurum*, *Stegonia*, and *Tortula*) (PP = 1; BS = 99) is placed sister to clade with the accessions currently considered as Syntrichieae (*Chenia*, *Hennediella*, *Sagenotortula*, *Stonea*, *Streptopogon*, *Syntrichia*, and *Willia*) (PP = 0.98). Relationships into the last clade are not resolved, and only a few lineages are well supported: the clade of *Stonea oleaginosa* and *Chenia leptophylla* (PP = 1; BS = 100), the clade with two species of *Hennediella* (PP = 1; BS = 100), and the *Syntrichia* core clade (PP = 1; BS = 99), that is clearly monophyletic only with *Sagenotortula*, *Streptopogon*, and *Willia* included, but paraphyletic with the well-supported *Sagenotortula quitoensis* as sister to the bulk of *Syntrichia* species with *Willia* nested, and the strongly supported clade of *Streptopogon*, *S. gemmascens*, and *S. amphidiacea*, in a basal position.

Like in the ITS analysis, relationships between *Syntrichia* species are poorly resolved, and strongly supported lineages coincide entirely in topology: the clade that includes the strongly grouped *Syntrichia costesii* and *S. serrulata* (PP = 1; BS = 100), the *S. ruralis* group with *Syntrichia caninervis*, *S. ruralis*, and *S. norvegica* (PP = 1; BS = 100), the strongly grouped *Syntrichia chisosa* and *S. obtusissima* (PP = 1; BS = 97), the clade of *S. bogotensis* and *S. angustifolia* with maximum support, and two samples of *Willia brachychaete* (PP = 1; BS = 100). *Syntrichia lithophila* and *S. magilliana* are sister with maximum support of the strongly supported clade of *S. percarnosa* and *S. breviseta* (PP = 1; BS = 100). The analysis also indicates strong support (PP = 0.98; BS = 91) for a monophyletic group composed of strongly grouped specimens of *Syntrichia gemmascens* (PP = 1; BS = 100), *S. amphidiacea* (PP = 1; BS = 100) as sister to *Streptopogon calymperes* but without support, and the strongly supported clade of *S. cavifolius* and *S. erythrodontus* (PP = 0.96; BS = 82), with *Syntrichia fragilis* as sister (PP = 0.98; BS = 88).

## 4. Discussion and Conclusions

The characters traditionally used to circumscribe the two propagulose species, *Syntrichia amphidiacea* and *S. gemmascens*, closely related to *Streptopogon*, have been analyzed through an intensive morphological study of specimens, as well as through analyzing much of the main descriptive literature on them. Our new molecular generated data show *Streptopogon* as monophyletic with *Syntrichia amphidiacea* and *S. gemmascens* included, in spite of the discrepancy of the ITS and plastid relationships, which could be evidence of an exchange of genetic material between species in various lineages of the Pottioideae. This position of *S. amphidiacea* and *S. gemmascens* is also supported by morphological data (i.e., costa anatomy, leaf shape, and asexual reproduction), and accepts the differentiation of laminal papillae and the presence of a stem central strand as new characters in the genus *Streptopogon.* In addition, we emphasize the anatomy of the costa as the main distinctive generic character in *Syntrichia*.

Our results suggest a minor realignment into Syntricheae on the basis of molecular and morphological data, although the relationships among lineages within this tribe are poorly resolved. Additionally, species delimitation using molecular data was consistent with our preliminary morphological inference.

*Sagenotortula* is a monospecific genus known from Mexico and the Andes of South America, characterized by stems with a central strand but lacking both a sclerodermis and hyalodermis (Figure 6A), leaves broadly lingulate to spathulate with margins plane and unbordered (Figure 6E), sometimes weakly dentate near the apex (Figure 6F), costa without a dorsal epidermis and with the dorsal stereid band poorly developed, usually only with substereids and with hydroids (Figure 6B1–B3), usually percurrent, laminal cells smooth and very large (Figure 6D), and basal cells weakly differentiated (Figure 6C). The sporophytes of *Sagenotortula* are like those of *Syntrichia*; see Zander [3] (pp. 272, Plate 113-1 and 10) and Mishler [49] (pp. 325, Figure 239-h-i). Some *Syntrichia* species have costae mostly with pseudostereids (Figure 1A), similar in appearance to that of *Sagenotortula*.

On the other hand, some species of *Syntrichia* show a cross-section of the costa in upper third of the leaf like that of *Sagenotortula*, as the stereids disappear completely (Figure 1B). In addition, others present a single laminal papilla only on the dorsal side or few and widely scattered laminal papillae (Figure 1I). The extreme variability of lamina papillosity and costa cell organization in *Syntrichia* supports the hypothetical accommodation of *Sagenotortula quitoensis* within *Syntrichia*. However, the significant reduction in the structure of the costa and stem, lacking stereids or sclerodermis, together with the complete absence of papillae in the lamina cells, support the circumscription of *Sagenotortula* as a distinct genus.

Brinda et al. [23] have recently proposed a new section for *Sagenotortula quitoensis* within *Syntrichia*. According to Zander [3], *Sagenotortula* could have immediate ancestors shared with *Syntrichia* on the basis of the loss of papillae and inflation of laminal cells. Our molecular results show *Sagenotortula* (Figure 3, Figure 4 and Figure 5) as the early divergent genus, sister to the main group of species of *Syntrichia.* We also prefer to accept *Sagenotortula* as a distinct genus very close to *Syntrichia*, highlighting its structural differentiation of the costa, completely without stereids (Figure 6B).

**Figure 7:**
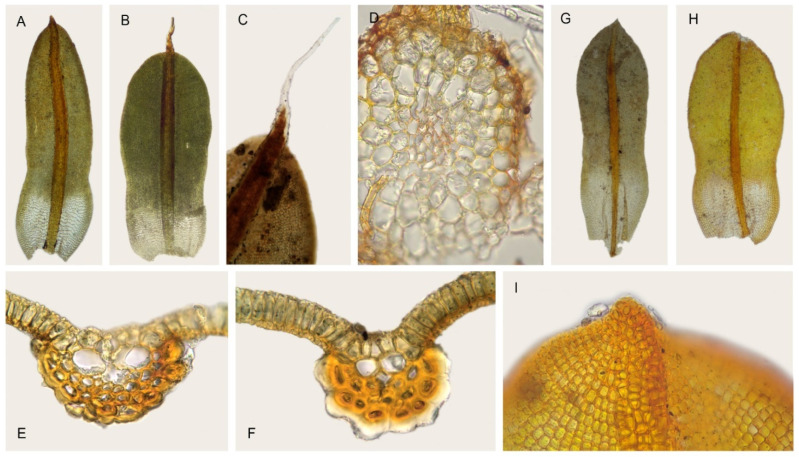
*Syntrichia breviseta*: (**A**) Stem leaf (MUB 52443); (**B**) Stem leaf (MUB 38283); (**C**) Detail of the pilose leaf apex (MUB 38283); (**D**) Cross-section of the stem (MUB 28735); (**E**) Cross-section of the costa at the middle of the leaf (MUB 38283); (**F**) Cross-section of the costa at the upper third (PC0052246). *Syntrichia percarnosa*; (**G**) Stem leaf (MUB 30092); (**H**) Stem leaf (MUB 40305); (**I**) Leaf apex, abaxial surface (MUB 40305). Photos: by M. Teresa Gallego.

In our analyses, *Syntrichia breviseta* and *S. percarnosa* form a separate clade closely sistered to *S. lithophila* and *S. magilliana* (Figure 3, Figure 4 and Figure 5), constituting a strongly supported and morphologically cohesive group. *Tortula breviseta* was described by Montagne [50] from Chilean material collected by Gay in 1829. Cano and Gallego [19] combined it with *Syntrichia*, highlighting the small size of its seta and other sporophytic characters (peristome with long spirally twisted teeth and short basal membrane). In the same work, these authors also synonymized the Chilean *Tortula pulvinatula* Dusén and *T. atrata* Thér. with *S. breviseta*, both with the distinguishing characters of the former; lingulate and constricted leaves, with plane or weakly recurved margins (Figure 7A,B), bordered by thicker walled and smooth cells, lamina irregularly bistratose, with a hyaline, short and smooth or spinulose hair-point (Figure 7B,C), upper and middle laminal cells 7.5–10 (12.5) µm wide, cross-section of the costa with hydroids and substereids (Figure 7E,F), and a weakly differentiated central strand in the stem (Figure 7D).

*Syntrichia lithophila* resembles *S. breviseta* in the constricted and apiculate leaves, with plane margins, although clearly differs in the propagulose leaf apex and unistratose leaf lamina. A detailed description of *S. lithophila*, as well as its differentiation from the nearby *S. sarconeurum* Ochyra & R.H. Zander can be found in Ochyra and Zander [16] and Ochyra et al. [51].

*Barbula percarnosa* Müll. Hal. was described by Müller [52] from subtropical Argentina. Later, Brotherus [53] transferred it to *Tortula*, and finally, Zander [3] combined it with *Syntrichia*. According to Gallego et al. [14], the Neotropical *Syntrichia percarnosa* has ovate-lingulate leaves, constricted in the middle (Figure 7G,H), with plane margins and differentiated borders, regularly bistratose or patchy bistratose lamina, middle laminal cells (5) 7.5–10 (12.5) µm wide, costa with hydroids, and a differentiated central strand. On the other hand, this taxon shares the presence of short dorsal costal cells in the upper third of the leaf (Figure 7I) with *S. breviseta*, which sometimes show a significant dorsal thickening, and are often hyaline (Figure 7F). Traditionally, they have been differentiated by the rounded and cucullate leaf apex in the epilose *S. percarnosa*, and the costa excurrent as a short hair-point in *S. breviseta*. Moreover, the former usually has smooth dorsal surface of the costa, while in *S. breviseta*, it is strongly mamillose.

After the study of hundreds of specimens from South America, it has been possible to verify the high variability of their distinguishing characters, even within individual specimens. Until recently, *Syntrichia breviseta* and *S. percarnosa* were considered to be two different species from South America [3,14,19,22], but our results reveal that both taxa should be treated as conspecific. We here propose to synonymize these two species on the basis of molecular and morphological data as *S. breviseta*, since this name has priority. Consequently, its distribution actually expands into the Neotropical area, as Gallego et al. [22] have already pointed out when citing for the first time *S. breviseta* from Bolivia.

The striking hyaline thickening of the dorsal cells of the costa (see Anderson [54] (pp. 16, Figure 6 and Figure 7), together with the constricted and apiculate leaves, with plane margins, probably justifies the presence of *S. magilliana* as a sister of the clade of *S. percarnosa* and *S. breviseta*, but the stems with undifferentiated central strands and unistratose leaves with unbordered margins characterize the species. Further study would help in the morphological characterization of this species, as to date, it has only been known from South Africa [54,55].

Our results agree with the recently established sect. *Vallidens* [23], including *S. breviseta*, *S. lithophila*, *S. magilliana*, *S. sarconeurum*, and *S. phaea*.

*Willia* is a closely related taxon to *Syntrichia*, sharing costal anatomy (Figure 1K) and lamina papillosity (Figure 1L), although differs by the reduction and complexity of its peristome, short setae, cucullate to long-mitrate calyptrae, and differentiated perichaetial leaves [51]. The mitrate calyptra is an unusual character within Pottiaceae, considered as apomorphic by Zander [3] and only differentiated in some genera of Pottioideae (*Acaulon* Müll. Hal., *Pterygoneurum* Jur., *Streptopogon*, and *Phascopsis* I.G. Stone). According to Zander [3], *Willia* may ultimately be accepted as a separate section into *Syntrichia*, highlighting the proximity of species with plane leaf margins of section *Aesiotortula*. Recently, Brinda et al. [23] have proposed a new section within *Syntrichia* to accommodate all species of *Willia*. In addition, they maintain the sect. *Aesiotortula* proposed by Zander [3], but in a different circumscription. We agree and consider that *Willia* should be included within *Syntrichia* on the basis of our morphological and molecular results.

**Figure 8:**
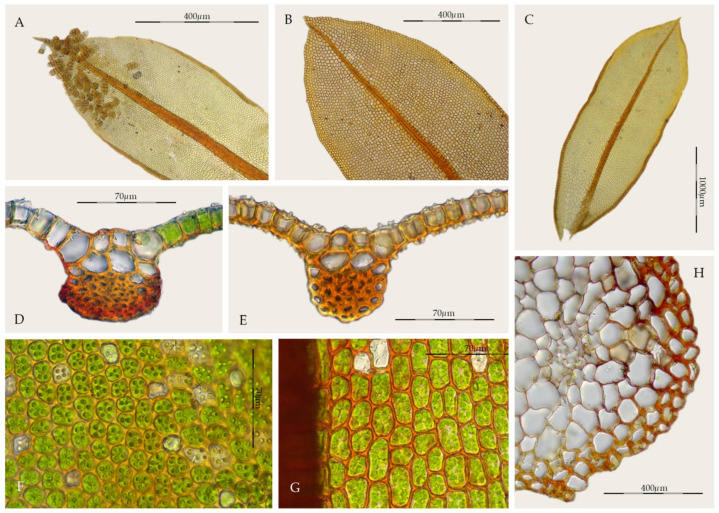
*Syntrichia amphidiacea*: (**A**) Propagulose leaf (MUB 56123); (**B**) Leaf apex (MUB 57451); (**C**) Stem leaf (MEXU 5008); (**D**) Cross-section of the costa at the middle of the leaf (MUB 57451); (**E**) Cross-section of the costa at the middle of the leaf (MEXU 2759); (**F**) Upper laminal cells (MEXU 5008); (**G**) Middle laminal cells (MUB 57451); (**H**) Cross-section of the stem (MUB 56123). Photos: by M. Teresa Gallego.

*Syntrichia amphidiacea* is characterized by lingulate-spathulate, lanceolate-sphathulate, or elliptical leaves (Figure 8C), not constricted in the middle, with acute, occasionally acuminate, and non-cucullate apices (Figure 8B), sometimes short apiculate, margins recurved two thirds of the length of the leaf (Figure 8A,C), unistratose and usually bordered, upper and mid-laminal cells rectangular to quadrate, with rounded corners, 12.5–27.5(32.5) × (10)12.5–17.5(22.5) μm, thin-walled, collenchymatous, and papillose (Figure 8F), although usually inconspicuously papillose or nearly smooth (Figure 8G), cross-section of the costa with 2–4 guide cells in 1 layer, with 4–9 dorsal stereid rows, without hydroids, sometimes with substereids (Figure 8D,E), juxtacostal basal cells hyaline, forming an inverted U-shaped group, although sometimes undifferentiated (Figure 8C), and stems lacking a hyalodermis, and with central strands weakly differentiated or undifferentiated (Figure 8H). However, the most distinctive character is the differentiation of multicellular propagula (laminar gemmae), borne on the ventral surface, usually also on the dorsal surface of the leaf on the lamina; they are cylindrical, claviform, 75–200 × 37.5–55 µm, sessile, green or brown, and smooth or weakly papillose (Figure 8A).

*Syntrichia amphidiacea*, is a dioicous species rarely producing sporophytes which are only known from southern Mexico [49]. All these characters traditionally place the taxon in *Syntrichia*, but the combination of the structure of the costa, with a semicircular dorsal stereid band in cross-section, sometimes only with substereids and the sometimes differentiated dorsal epidermis (Figure 8E), together with the type of propagules, really approximate it to *Streptopogon*. *Streptopogon* is traditionally distinguished from *Syntrichia* by its smooth laminal cells (Figure 2F), stem without a central strand (Figure 2G), a mitrate and papillose calyptra, the basal hyaline area weakly differentiated (Figure 2H), and the costa with a dorsal epidermis and a band of dorsal stereids in semicircular pattern (Figure 2D–E). We have studied several samples of *Syntrichia amphidiacea* without a central strand and others with poorly differentiated simple papillae on the lamina. In addition, *Streptopogon cavifolius*, *S. matudianus* H.A. Crum, and *S. lindigii* Hampe have glabrous calyptrae [3,26,28,56].

Without propagules, *Syntrichia amphidiacea* is very similar to *S. subpapillosa*, a species known from Chile [57] and Argentina [20]. Both species share the leaf shape, costa anatomy and bordered margins, but *S. subpapillosa* differs in the globose propagules, leaves with longer apiculi or hair-points, and, usually, with a more scarce laminal papillae.

The only discrepancy observed between ITS and plastid relationships, concerns the position of the Chinese *Syntrichia gemmascens*, which is nested within the Pottieae clade according to plastid information and within the *Streptopogon* clade according to ITS information. In this case, the morphology is consistent with the species suggested by nuclear information. The gene tree relating copies from various species might disagree with the species phylogeny [58]. It is difficult to assess which processes are causing this discrepancy between nuclear and plastid data, although incomplete lineage sorting or horizontal transfer (including hybridization) could explain it, since an exchange of genetic material between species would not be new in Pottiaceae [6,15,59].

**Figure 9:**
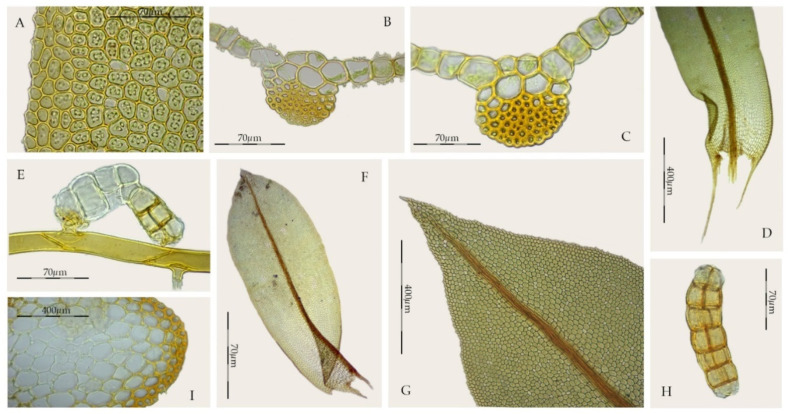
*Syntrichia gemmascens* (MUB 56121): (**A**) Marginal leaf cells; (**B**,**C**) Cross-section of the costa at the middle of the leaf; (**D**) Leaf base; (**E**) Propagule growing on rhizoid; (**F**) Stem leaf; (**G**) Leaf apex; (**H**) Propagule; (**I**) Cross-section of the stem. Photos: by M. Teresa Gallego.

*Syntrichia gemmascens* shares with *S. amphidiacea* the same type of propagules, although in the former they also grow on the rhizoids (Figure 9E), the cross-section of the costa with dorsal stereid band semicircular in shape and without hydroids (Figure 9B,C), the poorly differentiated laminal basal hyaline area (Figure 9D), the bordered margins (Figure 9A), the non-constriction at the middle of the leaves (Figure 9F), and the leaves with collenchymatous and papillose cells (although in *S. gemmascens*, the papillae are mainly bifurcate) (Figure 9A). In addition, the stems of *S. gemmascens* show a practically undifferentiated central strand, the margins at the leaf base are clearly decurrent (Figure 9D), are weakly recurved up to the middle of the leaf, and toothed distally (Figure 9G). Afonina and Ignatova [60] emphasized the close relationship of these two species, although they highlighted the differences between both taxa.

The clade of *Streptopogon*, *Syntrichia gemmascens*, and *S. amphidiacea* (Figure 3), strongly supported just with nuclear loci, forces a reinterpretation of the morphological generic boundaries of the genus *Streptopogon*, which has been traditionally recognized by smooth laminal cells, stems without central strand, and usually, scabrose and mitrate calyptrae [3,26,28], since two species of *Syntrichia* with papillose laminal cells, stems with central strand, and smooth and cucullate calyptrae are included in it. We therefore consider the differentiation of laminal papillae and the presence of a stem central strand as new characters in the genus *Streptopogon.*

Based on our molecular and morphological results, a diversification of both *Streptopogon* and *Syntrichia* can be interpreted, accepting the consequent update of their morphological distinctive characters at the generic level. We consider the lack of costal dorsal epidermis and the differentiation of a crescent-shaped costal dorsal stereid band as distinctive generic characters in *Syntrichia*, so, transferring *S. amphidiacea* and *S. gemmascens* to *Streptopogon* makes the former a morphologically more consistent genus. Therefore, we prefer to recognize *Streptopogon* as a distinct genus since it has a dorsal epidermis and a semicircular dorsal stereid band in the costa. The latter two characters, together with the differentiation of the same type of propagules, are some of the synapomorphies that support the placement of *S. amphidiacea* and *S. gemmascens* in the monophyletic *Streptopogon*.

We have not been able to find obvious morphological or anatomical synapomorphies to support the monophyly of *Syntrichia*, on the basis of our molecular results. On the other hand, the plastid analysis supports the monophyly of *Syntrichia*, only if *S. gemmascens* is removed from the genus. On the basis that molecular paraphyly explains much incongruence between morphological and molecular cladograms or other diagrams of evolutionary relationships, providing important information [24], we recognize *Syntrichia* (with *Willia* included), *Sagenotortula* and *Streptopogon* in a paraphyletic arrangement. In addition, this study emphasizes the need for a morphologically and geographically broad taxon sampling for a sound assessment of relationships with the Pottioideae.

## 5. Taxonomic Changes

Based on the molecular and morphological results presented in this study, we consider *Syntrichia amphidiacea* and *S. gemmascens* as species of *Streptopogon*, accepting their obvious morphological and molecular proximity, pending further studies that include all species of both genera over a wide geographical range. On the other hand, we consider *Syntrichia percarnosa* and *S. breviseta* as conspecific (as *S. breviseta*). The necessary nomenclatural changes and synonymies follow below. A worldwide taxonomic revision of the species belonging to *Syntrichia* is currently underway by the first two authors.

### 5.1. New Combinations in Streptopogon

***Streptopogon amphidiaceum*** (Müll. Hal.) M.T. Gallego & M.J. Cano, **comb. nov.** ≡ *Barbula amphidiacea* Müll. Hal., Linnaea 38: 639. 1874 ≡ *Tortula amphidiacea* (Müll. Hal.) Broth., Nat. Pflanzenfam. I (3): 434. 1902 ≡ *Syntrichia amphidiacea* (Müll. Hal.) R.H. Zander, Bull. Buffalo Soc. Nat. Sci. 32: 267. 1993—Type: Mexico. Monte Orizaba, *Müller s.n.* (not located, not at BM, JE, NY).

= *Tortula caroliniana* A.L. Andrews, Bryologist 23: 72. 5. 1920—Type: USA. North Carolina, Swannanoa River, Swannanoa, 9 July 1919, *A.L. Andrews 176* (**lectotype, designated here**: Andrews 176 CU!; isolectotypes NY00371644 and NY00371641).

= *Tortula subcaroliniana* Bizot, Svensk Bot. Tidskr. 63: 446. 1969—Type: Republic of Cabo Verde. Santo Antao Islands, 1958, *Byström s.n.* (PC-0702603).

= *Tortula tanganyikae* Dixon, J. Bot. 76: 252. 1938—Type: Tanzania. Mufindi, Tanganyca Territory, 1700 m, 2 May 1934, *G. Balbo 41* (holotype: BM-000729360!).

Nomenclatural note: *Tortula caroliniana* was described by Andrews [61] on the basis of his own material collected in North Carolina in 1919, growing on bark of deciduous trees. In the protologue, he mentioned three syntypes “bank of Swannanoa River, at Swannanoa, Buncombe Co., July 9; North Fork, some 5 miles above its confluence with Swannanoa River, July 10; Grandmother Gap, Avery Co., Aug 13”. In addition, he also thanks Dorothy Coker, of the New York Botanical Garden, for her drawings of *T. caroliniana*. We located three syntypes of this name in the author’s herbarium at CU. In NY, there are two duplicates of material collected in Swannanoa River on July 9 from Herbarium of A.L. Andrews (NY00371644 and NY00371641), being the first of these accompanied by the original illustration. As the author did not designate a holotype, all these specimens are syntypes [62] (Art. 40 Note 1) and a lectotypification is needed. We here choose as lectotype the specimen deposited at CU collected in Swannanoa River on July 9, because it was the one used for the illustration.

***Streptopogon gemmascens*** (P.C. Chen) M.T. Gallego, M.J. Cano & J.A. Jiménez **comb. nov.** ≡ *Desmatodon gemmascens* P.C. Chen, Hedwigia 80: 297. 1941 ≡ *Syntrichia gemmascens* (P.C. Chen) R.H. Zander, Bull. Buffalo Soc. Nat. Sci. 32: 269. 1993—Type: China. Prov. Yunnan, 3600–3700 m, 16 Sept 1915, *Handel-Mazzetti 8029* (holotype WU-0045789; isotype: H.BR-1282029!)

### 5.2. New Synonyms for Syntrichia

***Syntrichia breviseta*** (Mont.) M.J. Cano & M.T. Gallego, Bot. J. Linn. Soc. 156: 208. 2008 ≡ *Tortula breviseta* Mont., Ann. Sci. Nat., Bot., sér. 3, 4: 107. 1845 ≡ *Barbula breviseta* (Mont.) Müll. Hal., Syn. Musc. Frond. 1: 644. 1849—Type: Chile. S. Lago, *Gay s.n*. (**lectotype, designated here**: PC-0052237!; isolectotype: PC-0052236!).

= *Syntrichia percarnosa* (Müll. Hal.) R.H. Zander, Bull. Buffalo Soc. Nat. Sci. 32: 269. 1993, **syn. nov.** ≡ *Barbula percarnosa* Müll. Hal., Linnaea 42: 347. 1879 ≡ *Tortula percarnosa* (Müll. Hal.) Broth., Nat. Pflanzenfam. 1(3): 432. 1902—Type: Argentina. Argentina subtropica, Cuesta de Pinos, *Lorentz s.n.* (**lectotype, designated here**: S-B64118!).

Nomenclatural notes: *Tortula breviseta* was described by Montagne [50] on the basis of a single gathering collected in Chile. There are two specimens of this gathering housed in PC. Cano and Gallego [19] considered one of them as the holotype. However, this statement cannot be considered an inadvertent lectotypification, since the publication does not state that a lectotype is being designated and was published after 1 January 2001 (Art. 7.11 and Art. 9.23, Turland et al. [62]), and lectotypification is required. Here, material from PC (PC0052237) is selected as lectotype.

*Barbula percarnosa* was described by Müller [52] on the basis of material collected by P.G. Lorentz in Argentina. In the protolog, he mentioned a unique specimen “Argentinia subtropica, Cuesta de Pinos. Monte Nevado prope Salta, 11,000 ped.”. We located one syntype of this name in S, which we have chosen as the lectotype.

## Figures and Tables

**Table 1:** Statistics of the nuclear and chloroplast datasets analyzed in this study.

Locus	Number of Specimens	New Generated Sequences	Sequence Length	Parsimony-Informative Characters
ITS	48	35	1908	802 (42.03%)
*trn*G	43	22	647	91 (14%)
*trn*L-F	48	25	459	62 (13.50%)
Nuclear + plastid	51		3014	1213 (36.8%)

## Data Availability

DNA sequences are available on the GenBank database and all authors agree with MDPI Research Data Policies.

## Appendix A

Vouchers and GenBank accession numbers for taxa used in the molecular phylogenetic analysis. Taxon name, country and next division, voucher (collector, number, and where the specimen is housed), and GenBank accession number for DNA sequences ITS, *trn*L-F, and *trn*G; a dash (−) indicates missing data. An asterisk (*) is provided for sequences retrieved directly from GenBank.

***Bryoerythrophyllum recurvirrostrum*** (Hedw.) P.C. Chen: Spain, Cantabria, *Cano 4675* (MUB), MW398547*, GU953731*, GU953706*. ***Chenia leptophylla*** (Müll. Hal.) R.H. Zander: Venezuela, Caracas, *Cano & Jiménez 6073* (MUB), MW398561*, MW432560*, MW432806*. ***Crossidium squamiferum*** (Viv.) Jur.: Spain, Canary Islands, *Cano 4801* (MUB), MW398558*, JN968438*, JN968402*. ***Crumia latifolia*** (Kindb.) V.D. Schofield: USA, California, *Sagar 1672* (MUB), OM807138, KF418160*, KF418179*. ***Dolotortula mniifolia*** (Sull.) R.H. Zander: Argentina, Tucumán, *Cano* et al. *4238a* (MUB), MW398555*, MW432555*, MW432802*. ***Hennediella heimii*** (Hedw.) R.H. Zander: Bolivia, Oruro, *Cano & Jiménez 3745* (MUB), GQ339750*, KF418162*, KF418181*; ***Hennediella polyseta*** (Müll. Hal.) R.H. Zander: Ecuador, Chimborazo, *Cano 3093* (MUB), GQ339759*, KF418161*, KF418180*. ***Leptodontium excelsum*** (Sull.) E. Britton: Ecuador, Loja, *Cano & Gallego 3027* (MUB), MW398545*, GU953738*, GU953713*. ***Pterygoneurum ovatum*** (Hedw.) Dixon: Spain, Teruel, *Cano 5403* (MUB), MW398560*, MW432559*, –. ***Sagenotortula quitoensis*** (Taylor) R.H. Zander: **(1)** Chile, Providencia, *Larraín 41723* (MUB), OM807139, OM793089, –; **(2)** Bolivia, Oruro, *Cano* et al. *3416* (MUB), GQ339761*, MW432562*, MW432808*. ***Stegonia latifolia*** (Schwägr.) Venturi ex Broth.: Spain, Burgos, *Cano 9570* (MUB), MW398559*, MW432558*, MW432805*. ***Stonea oleaginosa*** (I.G. Stone) R.H. Zander: Australia, Victoria, *Holley s.n.* (MUB), OM807140, OM793090, OM793067. ***Streptopogon calymperes*** Müll. Hal.: Venezuela, Mérida, *Grande, Cano & Jiménez 5941a* (MUB), OM807141 (ITS1), OM793091, OM793068; ***Streptopogon cavifolius*** Mitt.: **(1)** Nicaragua, Estelí, *Cano & Alonso 9663c* (MUB), –, –, OM793069; **(2)** Mexico, *Burghardt 4359* (MEXU), –, OM793092, –; ***Streptopogon erythrodontus*** (Taylor) Wilson ex Mitt.: **(1)** Venezuela, Trujillo, *Cano & Jiménez 5784* (MUB), OM807142, OM793093, OM793070; **(2)** Perú, La Libertad, *Cano & Jiménez 5336* (MUB), OM807143, OM793094, OM793071; **(3)** Bolivia, La Paz, *Schäefer-Verwimp & Verwimp 11903* (CAS), OM807144 (ITS1), –, –. ***Syntrichia amphidiacea*** (Müll. Hal.) R.H. Zander: **(1)** Bolivia, Santa Cruz, *Churchill 20723* (MO), OM807145, OM793095, OM793072; **(2)** Cabo Verde, Santiago, *Cano 7968* (MUB), OM807146, OM793096, OM793073; **(3)** Venezuela, Mérida, *Grande, Cano & Jiménez 5941b* (MUB), OM807147, OM793097, OM793074; ***Syntrichia*** ***angustifolia*** (Herzog) M.J. Cano: Bolivia, La Paz, *Fuentes, Jiménez & Quisbert 12002* (MUB), OM807148, OM793098, OM793075; ***Syntrichia*** ***bogotensis*** (Hampe) Mitt. ex R.H. Zander: Ecuador, Pichincha, *Cano* et al. *2758a* (MUB), OM807149, KF417176*, KF418195*; ***Syntrichia*** ***breviseta*** (Mont.) M.J. Cano & M.T. Gallego: **(1)** Argentina, Catamarca, *Cano* et al. *4150* (MUB), OM807150, OM793099, OM793076; **(2)** Bolivia, Tarija, *Linneo 930* (MUB), –, OM793100, OM793077; ***Syntrichia*** ***caninervis*** Mitt.: Spain, Murcia, *López s.n.* (MUB), OM807151, KF418173*, KF418192*; ***Syntrichia*** ***chisosa*** (Magill., Delgad. & L.R. Stark) R.H. Zander: Argentina, La Rioja, *Cano* et al. *4338* (MUB), OM807152, OM793101, OM793078; ***Syntrichia costesii*** (Thér.) R.H. Zander: Chile, Los Lagos, *Cano 588a* (MUB), OM807153, KF418167*, KF418186*; ***Syntrichia fragilis*** (Taylor) Ochyra: Ecuador, Loja, *Cano & Gallego 3002* (MUB), OM807154, KF418174*, KF418193*; ***Syntrichia gemmascens*** (P.C. Chen) R.H. Zander: **(1)** China, Yunnan, *Shevock 45595* (MUB), OM807155, OM793102, OM793079; **(2)** China, Yunnan, *Shevock 45651* (MUB), OM807156, OM793103, OM793080; **(3)** China, Xun-Dian, *Shevock 50460* (MUB), OM807157, OM793104, OM793081; **(4)** China, Shangri-La, *Shevock 50581* (MUB), OM807158, OM793105, OM793082; ***Syntrichia kingii*** (H. Rob.) M.T. Gallego & M.J. Cano: Peru, Cajamarca, *Cano* et al. *5089* (MUB), OM807159, KF418165*, KF418184*; ***Syntrichia lithophila*** (Dusén) Ochyra & R.H. Zander: Chile, Magallanes, *Cano 665b* (MUB), OM807160, OM793106, –; ***Syntrichia magellanica*** (Mont.) R.H. Zander: Chile, Valparaíso, *Cano 88* (MUB), OM807161, OM793107, –; ***Syntrichia magilliana*** L.E. Anderson: South Africa, Western Cape, *Hedderson 15364* (MUB), OM807162, –, –; ***Syntrichia norvegica*** F. Weber: Romania, Argues, *Cano* et al. *5419* (MUB), OM807163, KF418168*, KF418187*; ***Syntrichia obtusissima*** (Müll. Hal.) R.H. Zander: Argentina, Tucumán, *Cano* et al. *4039* (MUB), OM807164, OM793108, OM793083; ***Syntrichia papillosa*** (Wilson ex Spruce) Spruce: Romania, Dambovita, *Cano* et al. *5432* (MUB), OM807165, OM793109, OM793084; ***Syntrichia percarnosa*** (Müll. Hal.) R.H. Zander: **(1)** Argentina, La Rioja, *Cano* et al. *4298* (MUB), OM807166, KF418170*, KF418189*; **(2)** Argentina, San Juan, *Cano* et al. *4358* (MUB), OM807167, KF418171*, KF418190*; ***Syntrichia ruralis*** (Hedw.) F. Weber & D. Mohr: Spain, Zamora, *Guerra* et al. *s.n.* (MUB), MW398564*, GU953737*, GU953712*; ***Syntrichia serripungens*** (Lorentz & Müll. Hal.) R.H. Zander: Argentina, Tucumán, *Cano* et al. *4191* (MUB), OM807168, OM793110, OM793085; ***Syntrichia serrulata*** Warnst.: Chile, Magallanes, *Cano* et al. *651a* (MUB), OM807169, KF418163*, KF418182*. ***Tortula atrovirens*** (Sm.) Lindb.: Spain, Murcia, *Cano* et al. *4166* (MUB), OM807170, OM793111, OM793086; ***Tortula muralis*** Hedw.: Spain, Pontevedra, *Cano 4524* (MUB), MW398562*, GU953736*, GU953711*; ***Tortula subulata*** Hedw.: Spain, Málaga, *Cabezudo* et al. *s.n.* (MUB), MW398563*, MW432561*, MW432807*. ***Willia brachychaete*** (Dusén) R.H. Zander **(1)** Chile, La Araucanía, *Cano 485* (MUB), OM807171, OM793112, OM793087; **(2)** Chile, Bío-Bío, *Ireland & Bellolio 35711* (MUB), OM807172, OM793113, OM793088.

## References

[1] Zander R.H. (2006). The Pottiaceae s. str. as an evolutionary Lazarus taxon. J. Hattori Bot. Lab..

[2] Frey W., Stech M., Frey W. (2009). Marchantiophyta, Bryophyta, Anthocerotophyta. Syllabus of Plant Families, A. Engler’s Syllabus der Pflanzenfamilien.

[3] Zander R.H. (1993). Genera of the Pottiaceae: Mosses of harsh environments. Bull. Buffalo Soc. Nat. Sci..

[4] Goffinet B., Buck W.R., Shaw A.J., Goffinet B., Shaw A.J. (2009). Morphology, anatomy, and classification of the Bryophyta. Bryophyte Biology.

[5] Saito K. (1975). A monograph of Japanese Pottiaceae (Musci). J. Hattori Bot. Lab..

[6] Cano M.J., Jiménez J.A., Gallego M.T., Guerra J. A molecular approach to the phylogeny of the moss genus *Pseudocrossidium* (Pottiaceae, Bryopsida) and its taxonomic implications. J. Syst. Evol..

[7] Gallego M.T., Hugonnot V., Cano M.J. (2018). Taxonomic resurrection of an awnless variety of *Syntrichia ruralis* and comparison with other European muticous taxa in this genus. J. Bryol..

[8] Gallego M.T., Cano M.J. (2021). *Syntrichia splendida* M.T.Gallego & M.J.Cano (Pottiaceae), a new moss species from northern Chile. J. Bryol..

[9] Zander R.H. (1989). Seven new genera in Pottiaceae (Musci) and a lectotype for *Syntrichia*. Phytologia.

[10] Ochyra R. (1992). New combinations in *Syntrichia* and *Warnstorfia* (Musci). Fragm. Florist. Geobot..

[11] Spagnuolo V., Caputo P., Cozzolino R., Castaldo R., De Luca P. (1999). Patterns of relationships in Trichostomoideae (Pottiaceae, Musci). Plant Syst. Evol..

[12] Werner O., Ros R.M., Cano M.J., Guerra J. (2002). *Tortula* and some related genera (Pottiaceae, Musci): Phylogenetic relationships based on chloroplast rps4 sequences. Pl. Syst. Evol..

[13] Afonina O.M., Ignatova E.A., Fedosov V.E., Kuznetsova O.I. (2014). Toward a new understanding of *Syntrichia submontana* (Pottiaceae, Bryophyta). Arctoa.

[14] Gallego M.T., Cano M.J., Jiménez J.F., Jiménez J.A., Guerra J. (2014). Morphological and molecular data support a new combination in the Neotropical complex of cucullate-leaved species of *Syntrichia* (Pottiaceae). Syst. Bot..

[15] Hedenäs L., Heinrichs J., Gallego M.T. (2019). The Scandinavian *Syntrichia ruralis* complex (Musci, Pottiaceae): A chaos of diversification. Plant Syst. Evol..

[16] Ochyra R., Zander R.H. (2007). Is *Tortula lithophila* conspecific with *Sarconeurum glaciale* (Bryopsida: Pottiaceae)?. Frag. Florist. Geobot. Polon..

[17] Cano M.J. (2007). Typification and taxonomical identity of some infraspecific name related to *Tortula subulata* complex (Pottiaceae, Bryophyta). Taxon.

[18] Cano M.J. (2008). Taxonomic revision of *Hennediella* Paris (Pottiaceae, Bryophyta). Nova Hedwig..

[19] Cano M.J., Gallego M.T. (2008). The genus *Tortula* (Pottiaceae, Bryophyta) in South America. Bot. J. Linn. Soc..

[20] Matteri C.M. (2003). New combination and new synonyms in Fuegian mosses. Lindbergia.

[21] Gallego M.T., Cano M.J., Guerra J. (2009). New synonymy in *Syntrichia* (Pottiaceae) in the Neotropics. Bryologist.

[22] Gallego M.T., Cano M.J., Guerra J. (2011). New records, synonyms and one combination in the genus *Syntrichia* (Pottiaceae) from South America. Bryologist.

[23] Brinda J.C., Jáuregui-Lazo J.A., Oliver M.J., Mishler B.D. (2021). Notes on the genus *Syntrichia* with a revised infrageneric classification and the recognition of a new genus *Syntrichiadelphus* (Bryophyta, Pottiaceae). Phytologia.

[24] Zander R.H. (2019). Macroevolutionary versus molecular analysis: Systematics of the *Didymodon* segregates *Aithobryum*, *Exobryum* and *Fuscobryum* (Pottiaceae, Bryophyta). Hattoria.

[25] Pottiaceae, Integrated Taxonomic Information System. http://www.pottiaceae.com/index.php?mod=field_trips.

[26] Casado C.M. (2000). A Taxonomic Revision of Streptopogon Wils. (Pottiaceae), Thesis.

[27] Costa D.P. (2012). Validation of the new species of *Streptopogon* (Pottiaceae, Bryophyta) and a synopsis of the genus for Brazil. Syst. Bot..

[28] Salmon E.S. (1903). A monograph of the genus *Streptopogon*, Wils. Ann. Bot..

[29] Jiménez J.A., Cano M.J., Guerra J. (2021). A multilocus phylogeny of the moss genus *Didymodon* and allied genera (Pottiaceae): Generic delimitations and their implications for systematics. J. Syst. Evol..

[30] Doyle J.J., Doyle J.L. (1987). A rapid DNA isolation procedure for small quantities of fresh leaf tissue. Phytochem. Bull..

[31] Suzuki T., Inoue Y., Tsuba H., Iwatsuki Z. (2013). Notes on *Aptychella* (Sematophyllaceae, Bryopsida): *Yakushimabryum longissimum*, syn. nov. Hattoria.

[32] Cano M.J., Jiménez J.F., Gallego M.T., Jiménez J.A., Guerra J. (2009). Phylogenetic relationships in the genus *Hennediella* (Pottiaceae, Bryophyta) inferred from nrITS sequence data. Pl. Syst. Evol..

[33] Pacak A., Szweykowska-Kulińska Z. (2000). Molecular data concerning the allopolyploid character and the origin of chloroplast and mithochondrial genomes in the liverwort species *Pellia borealis*. J. Pl. Biotech..

[34] Taberlet P., Gielly L., Pautou G., Bouvet J. (1991). Universal primers for amplification of three non-coding regions of chloroplast DNA. Pl. Mol. Biol..

[35] Stech M., Frahm J.P. (1999). The status of *Platyhypnidium mutatum* Ochyra & Vanderpoorten and the systematic value of Donrichardsiaceae based on molecular data. J. Bryol..

[36] Sawicki J., Szczecińska M. (2011). A comparison of PCR-based markers for molecular identification of *Sphagnum* species of the section *Acutifolia*. Acta Soc. Bot. Pol..

[37] Olsson S., Buchbender V., Enroth J., Hedenäs L., Huttunen S., Quandt D. (2009). Phylogenetic analyses reveal high levels of polyphyly among pleurocarpous lineages as well as novel clades. Bryologist.

[38] Kearse M., Moir R., Wilson A., Stones-Havas S., Cheung M., Sturrock S., Buxton S., Cooper A., Markowitz S., Duran C. (2012). Geneious basic: An integrated and extendable desktop software platform for the organization and analysis of sequence data (version 9.1.8). Bioinformatics.

[39] Edgar R.C. (2004). MUSCLE: Multiple sequence alignment with high accuracy and high throughput. Nucl. Acids Res..

[40] Müller K. (2005). SeqState: Primer design and sequence statistics for phylogenetic DNA data sets. Appl. Bioinform..

[41] Simmons M.P., Ochoterena H. (2000). Gaps as characters in sequence based phylogenetic analyses. Syst. Biol..

[42] Darriba D., Taboada G.L., Doallo R., Posada D. (2012). jModelTest 2: More models, new heuristics and parallel computing. Nat. Methods.

[43] Tamura K., Nei M. (1993). Estimation of the Number of Nucleotide Substitutions in the Control Region of Mitochondrial DNA in Humans and Chimpanzees. Mol. Biol Evol..

[44] Stamatakis A. (2014). Raxml version 8: A tool for phylogenetic analysis andpost-analysis of large phylogenies. Bioinformatics.

[45] Edler D., Klein J., Antonelli A., Silvestro D. (2020). raxmlGUI 2.0 beta: A graphical interface and toolkit for phylogenetic analyses using RAxML. bioRxiv.

[46] Ronquist F., Teslenko M., van der Mark P., Ayres D.L., Darling A., Höhna S., Larget B., Liu L., Suchard M.A., Huelsenbeck J.P. (2012). MrBayes 3.2: Efficient bayesian phylogenetic inference and model choice across a large model space. Syst. Biol..

[47] Miller M.A., Pfeiffer W., Schwartz T. The CIPRES science gateway: Enabling high-impact science for phylogenetics researchers with limited resources. Proceedings of the Conference of the Extreme Science and Engineering Discovery Environment: Bridging from the Extreme to the Campus and Beyond, Association for Computing Machinery.

[48] Rambaut A. FigTree, Version 1.4.4. http://tree.bio.ed.ac.uk/software/figtree/.

[49] Mishler B.D., Sharp A.J., Crum H., Eckel P.M. (1994). Tortula. The Moss Flora of Mexico.

[50] Montagne C. (1845). Cinquième centurie de plantes cellulaires exotiques nouvelles. Ann. Sci. Nat. Bot. Sér. 3.

[51] Ochyra R., Lewis Smith R.I., Bednarek-Ochyra H. (2008). The Illustrated Moss Flora of Antarctica.

[52] Müller C. (1879). Prodromus bryologiae Argentinicae. I. Linnaea.

[53] Brotherus V.F., Engler H.G.A., Prantl K.A.E. (1902). Bryales. Die Natürlichen Pflanzenfamilien.

[54] Anderson L.E. (1997). *Syntrichia magilliana* (Pottiaceae), a new species from South Africa. J. Hattori Bot. Lab..

[55] Van Rooy J., Perold S.M., Meyer N.L., Steenkamp Y., Keith M. (2006). Bryophyta. A Checklist of South African plants, Germishuizen, G..

[56] Crum H.A. (1952). Two new species of Mexican mosses collected by Aaron, J. Sharp. Bryologist.

[57] Müller F. (2009). An updated checklist of the mosses of Chile. Arch. Bryol..

[58] Maddison W.P. (1997). Gene trees in species trees. Syst. Biol..

[59] Košnar J., Herbstová M., Kolář F., Koutecký P., Kučera J. (2012). A case of intragenomic ITS variation in bryophytes: Assessment of gene flow and role of plyploidy in the origin of European taxa of the *Tortula muralis* (Musci: Pottiaceae) complex. Taxon.

[60] Afonina O.M., Ignatova E.A. (2009). *Syntrichia amphidiacea* (Pottiaceae), a new species for the moss flora of Russia. Bot. Zhur. (St. Petersburg).

[61] Andrews A.L. (1920). *Tortula caroliniana*, new species. Bryologist.

[62] Turland N.J., Wiersema J.H., Barrie F.R., Greuter W., Hawksworth D.L., Herendeen P.S., Knapp S., Kusber W.H., Li D.Z., Marhold K. (2018). International Code of Nomenclature for Algae, Fungi, and Plants (Shenzhen Code) Adopted by the Nineteenth International Botanical Congress Shenzhen, China, July 2017, Regnum Veg..

